# Surgical outcomes of pulmonary mucoepidermoid carcinoma: A review of 41 cases

**DOI:** 10.1371/journal.pone.0176918

**Published:** 2017-05-02

**Authors:** Chih-Cheng Hsieh, Yung-Han Sun, Shih-Wei Lin, Yi-Chen Yeh, Mei-Lin Chan

**Affiliations:** 1 Division of Thoracic Surgery, Department of Surgery, Taipei Veterans General Hospital, Taipei, Taiwan; 2 Institute of Clinical Medicine and Department of Surgery, School of Medicine, National Yang-Ming University, Taipei, Taiwan; 3 Graduate Institute of Business and Management, Chang Gung University, Taoyuan City, Taiwan; 4 Department of Information Management, Chang Gung University, Taoyuan City, Taiwan; 5 Department of Industrial Engineering and Management, Ming Chi University of Technology, Taipei, Taiwan; 6 Stroke Center and Department of Neurology, Linkou Chang Gung Memorial Hospital, Taoyuan City, Taiwan; 7 Department of Pathology and Laboratory Medicine, Taipei Veterans General Hospital, Taipei, Taiwan; 8 Thoracic Surgery Division, Surgery Department, Mackay Memorial Hospital, Taipei, Taiwan; Fu Jen Catholic University, TAIWAN

## Abstract

**Introduction:**

Pulmonary mucoepidermoid carcinoma is a rare cancer that occurs primarily in younger patients. The prognostic factors of pulmonary mucoepidermoid carcinoma are largely undetermined, especially in elderly patients. The aim of this study was to examine the clinical characteristics and prognostic factors influencing survival after surgical resection in patients with pulmonary mucoepidermoid carcinoma and also analyze the clinical manifestations and prognostic factors in elderly patients.

**Materials and methods:**

The pathological records of 41 pulmonary mucoepidermoid carcinoma patients (mean age, 61.4 years) who underwent surgical resection at our hospital between January 1991 and July 2015 were retrospectively reviewed. Subjects >65 years of age (n = 22) were considered elderly.

**Results:**

The median follow-up duration was 42.9 (interquartile range, 15.0–120.8) months. Sixteen patients (39.0%) experienced tumor relapse, including 13 patients (81.3%) within 2 years. The 5-year disease-free survival rate was 57.9%. Tumor grade did not influence disease-free survival (P = 0.286). In the multivariate analysis, age, tumor size, pathological T3–4 status, and pathological N2 status were independent predictors of disease-free survival. The 5-year overall survival rate was 57.0%. Tumor grade also did not influence overall survival (P = 0.170). Age, tumor size, pathological T status, and pathological N2 status were independent predictors of overall survival. In elderly patients, the 5-year disease-free survival and overall survival rates were 41.4% and 41.5%, respectively. Pathological T status was the only independent predictor of both disease-free survival and overall survival in elderly patients.

**Conclusions:**

Prognostic factors identified for pulmonary mucoepidermoid carcinoma in this study differ from those of previous studies. Principally, tumor grade did not influence either disease-free survival or overall survival. Age, tumor size, and pathological factors were independent predictors of disease-free survival and overall survival. In elderly patients, pathological T status was the only independent predictor of disease-free survival and overall survival.

## Introduction

Salivary gland cancer has a relatively low incidence, accounting for approximately 6% of all head and neck cancers in the United States [1]. Salivary gland-type malignancies have a similar histological appearance to salivary gland cancer. However, they arise from non-salivary gland locations. The lungs represent the most common extra-salivary gland site, due to the presence of submucosal glands that line the tracheobronchial tree [2–5]. Mucoepidermoid carcinoma and adenoid cystic carcinoma are the principal histological subtypes of salivary gland-type lung cancer [4, 6].

Pulmonary mucoepidermoid carcinoma (PMEC) is a rare tumor that accounts for 0.1–0.2% of all pulmonary cancers and only few reports have been published in the literature [2, 7, 8]. PMEC is now defined as a tumor characterized by a combination of squamous, mucus-secreting, and intermediate cell types, and is usually classified into low-grade and high-grade PMEC, according to histological appearance, mitotic activity, cellular atypia, local invasion, and necrosis. Histological grade, cancer stage, and lymph node metastasis are all reported to be independent prognostic factors of PMEC [9, 10]. Age, sex, tumor size, and other clinical characteristics have also been reported to influence survival in patients with PMEC [10–13].

Although PMEC has been reported in patients from 3–78 years of age, some studies [10–16] have shown that it most frequently occurs in younger adults with an average age of <50 years. However, in our experience and the experience of others [17, 18], the majority of patients are older than 50 years of age. The relationships between the clinical behavior, prognosis, and tumor histology in older patients have yet to be determined. The aim of this study was to examine the clinical characteristics and prognostic factors influencing survival after surgical resection in patients with PMEC and also to analyze the clinical manifestations and prognostic factors in older patients.

## Materials and methods

This study was approved by the Institutional Review Board committee of Taipei Veterans General Hospital. Patient informed consent was waived due to the retrospective nature of the study. Research was conducted in accordance with the 1964 Declaration of Helsinki and its later amendments.

Pathological records were identified from a prospectively collected database using text mining techniques. The medical records of patients treated for PMEC at the Division of Thoracic Surgery at Taipei Veterans General Hospital between January 1991 and July 2015 were retrospectively reviewed. Patients who were <18 years of age, who had received non-surgical treatment or biopsy only, and had a final pathological diagnosis other than PMEC were excluded. All of the specimens were reviewed by a pulmonary pathologist who confirmed the diagnosis and histological grading was performed according to the 2015 World Health Organization’s Classification of Tumours of the Lung, Pleura, Thymus, and Heart (fourth edition) [5]. The majority of the patients underwent preoperative examinations, including bronchoscopy, computed tomography, and whole body nuclear scanning, to determine the clinical stage according to the Union for International Cancer Control/American Joint Committee on Cancer TNM staging system (seventh edition) [19]. Follow-up studies, including biochemical testing, computed tomography scanning, and nuclear imaging, were performed every 3–6 months for the first 5 years and annually thereafter. Disease-free survival (DFS) was calculated from the date of first treatment to the date of the first relapse (recurrence or metastasis). Overall survival (OS) was calculated from the date of first treatment to the date of death from any cause or last follow-up. Subjects >65 years of age (n = 22) were considered elderly.

### Statistical analyses

Categorical variables were analyzed using the Spearman correlation test. Continuous variables were subgrouped using the median value for survival estimation. Survival curves were estimated using the Kaplan-Meier method in patient subgroups by categorical variables or regrouped continuous variables and compared using the log-rank test. Variables with a P < 0.10 in the univariate analysis were included in the multivariate analysis. Significance levels and estimates of hazard ratios and 95% confidence intervals were determined using a stepwise Cox regression model. All statistical analyses were conducted using Statistical Package for the Social Sciences for Windows, software version 18.0 (SPSS Inc., Chicago, IL, USA). A P < 0.05 was considered statistically significant.

## Results

**Fig 1:**
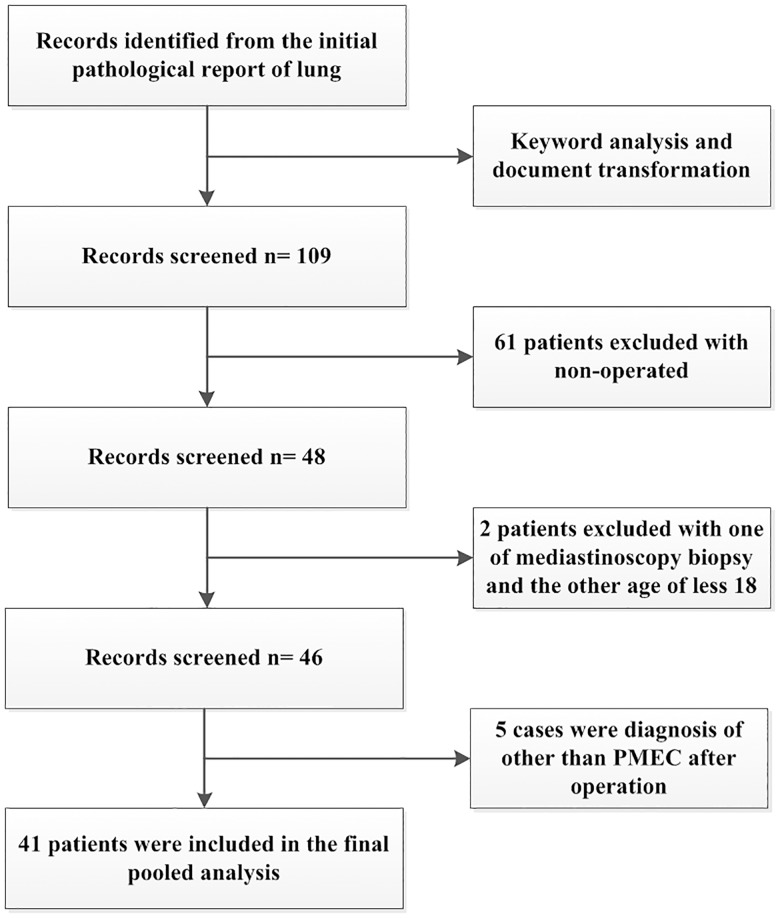
Patient flow diagram.

From the pathology database, 109 patients were identified as having a diagnosis of PMEC. Sixty-one patients (56.0%) were excluded due to non-surgical treatment methods, one patient (0.9%) underwent staging mediastinoscopy only, one patient (0.9%) was only 3 years of age, and 5 patients (4.6%) were excluded because they had a postoperative diagnosis other than PMEC (Fig 1). The remaining 41 patients (37.6%) had undergone curative resection and were included in this study. Of the 5,604 patients who underwent surgical resection for lung cancer at our hospital between January 1991 and July 2015, PMECs accounted for 0.7% (41/5,604) of all resected pulmonary malignancies during the 24-year study period.

Of the 41 patients included in this study, 30 patients (73.2%) were male and 11 (26.8%) were female. The mean age of the patients was 61.4 (range, 18–87) years. Male patients were significantly older than female patients (mean age: 67.2 vs. 45.2 years; P = 0.001). Twenty-one patients (51.2%) had a history of smoking with a smoking index of 10–100 pack-years. Cough was the most common presenting symptom (n = 23 patients; 56.1%) and was combined with hemoptysis in 8 patients (19.5%), blood-tinged sputum in 3 patients (7.3%), and whitish sputum in 6 patients (14.6%). Chest pain was the major presenting symptom in 4 patients (9.8%).

Bronchoscopy was performed in the majority of the patients. Abnormal findings, ranging from bronchial orifice narrowing to tumor obstruction, were noted in 16 patients (39.0%). Most of the patients (n = 29; 70.7%) underwent preoperative diagnostic assessment, including bronchoscopic biopsy in 16 patients (55.2%), computed tomography-guided biopsy in 10 patients (34.5%), and sputum cytological analysis in 3 patients (10.3%). With the exception of 8 patients (27.6%) without a diagnosis of malignancy, 15 patients (51.7%) were diagnosed with non-small cell lung cancer (squamous cell carcinoma [n = 4] and adenocarcinoma [n = 2]). Only 6 patients (20.7%), including 4 patients (66.7%) who were diagnosed by bronchoscopic biopsy, had a precise preoperative diagnosis of PMEC.

The majority of the patients (n = 30; 73.2%) underwent open thoracotomy because video-assisted thoracoscopic surgery was introduced late in the study period. Five patients (12.2%) underwent wedge resection or segmentectomy only due to poor pulmonary function. Lobectomy was performed in 31 patients (75.6%). Of these, 2 patients underwent sleeve lobectomy. Five patients (12.2%) underwent a more extensive resection than lobectomy (pneumonectomy [n = 4] and sleeve bilobectomy [n = 1]) due to the location of the tumor location. Seven patients (17.1%) had complications primarily involving persistent air leakage, one patient (2.4%) had atelectasis, and one patient (2.4%) had pneumonia. There were no cases of surgical mortality.

According to the Union for International Cancer Control/American Joint Committee on Cancer TNM staging system (seventh edition) [19], 13 patients (31.7%) had pathological (p) T1, 24 patients (58.6%) had pT2, 3 patients (7.3%) had pT3, and one patient (2.4%) had pT4 lesions. The median tumor size was 3.0 (range, 0.8–10.0) cm, and tumor size correlated with pT status (P = 0.016). The mean number of resected lymph nodes was 20.3 (range, 5–50). Twenty-six patients (63.4%) had pN0, 6 patients (14.6%) had pN1, and 9 patients (22.0%) had pN2 disease. Of the 9 patients with pN2 disease, only one patient (11.1%) had preoperative clinical N2 disease and none of these 9 patients received induction therapy prior to surgery. Twenty-one patients (51.2%) had pStage I, 11 patients (26.8%) had pStage II, 7 patients (17.1%) had pStage III, and 2 patients (4.9%) had pStage IV disease, with pStage correlating with pT and pN status. Ten patients (24.4%) had low-grade tumors; the remaining 31 patients (75.6%) had high-grade tumors. Patients with low-grade tumors were significantly younger than those with high-grade tumors (mean age: 42.4 vs. 67.5 years; P < 0.001). Angiolymphatic invasion (ALI) was present in 11 patients (26.8%) and correlated with pT status (P = 0.048). Pleural invasion was observed in 17 patients (41.5%). Sixteen patients (39.0%) received postoperative adjuvant treatment for locally advanced disease or lymph node metastasis based on pathological findings.

The median follow-up duration was 42.9 (interquartile range, 15.0–120.8) months. By the end of the study period, 16 patients (39.0%) experienced tumor relapse, including 13 patients (81.3%) within the first 2 years. The 3- and 5-year DFS rates were 65.9% and 57.9%, respectively. The univariate and multivariate analyses of DFS are presented in Table 1 with detailed information provided in S1 Table. Age <65 years, a small tumor size, and no postoperative adjuvant treatment were associated with a better DFS. Disease stage, pT status, and pN status influenced DFS, whereas tumor grade did not (P = 0.286). In the multivariate analysis, age, tumor size, pT3–4 status, and pN2 status were independent prognostic factors for DFS.

**Table 1:** Univariate and multivariate analyses of prognostic factors influencing disease-free survival (DFS) after surgical resection in all patients combined (n = 41) and in elderly patients (n = 22).

Factor	All Patients Combined					Elderly Patients (>65 y)				
	Univariate			Multivariate		Univariate			Multivariate	
	n	5-y DFS	P-value	HR (95% CI)	P-value	n	5-y DFS	P-value	HR (95% CI)	P-value
**Age (y)**			0.012*	1.10 (1.03–1.18)	0.003*	–	–	–	–	–
≤65	19	74.9%				–	–	–	–	–
>65	22	41.4%				–	–		–	
**Tumor Size (cm)**			0.005*		0.005*			0.082		0.076
≤3	25	72.8%		1		14	49.5%		1	
>3	16	32.8%		6.53 (1.78–24.0)		8	28.6%		3.17 (0.89–11.4)	
**pT Status**			0.054					<0.001*		
T1	13	82.5%		1		6	66.7%		1	
T2	24	53.5%		3.22 (0.49–21.0)	0.221	15	37.7%		7.72 (1.47–40.5)	0.016*
T3–4	4	25.0%		22.5 (1.10–461.9)	0.043*	1	0.0%		93.9 (4.25–2,073.6)	0.004*
**pN Status**			0.021*					0.331		
N0	26	68.5%		1		16	46.5%		–	
N1	6	66.7%		5.81 (0.77–44.1)	0.089	1	0.0%		–	
N2^a^	9	0.0%		12.0 (2.63–54.7)	0.001*	5	40.0%		–	–
**pStage**			0.015*					0.289		
I	21	69.8%		1		13	49.1%		–	
II	11	63.6%		2.12 (0.33–13.8)	0.432	4	25.0%		–	
III–IV^a^	9	0.0%		12.0^a^ (2.63–54.7)	0.001*	5	40.0%		–	–
**Tumor Grade**			0.286					0.146		
Low	10	66.7%		–	–	1	0.0%		–	–
High	31	54.9%		–		21	43.6%		–	
**ALI**			0.145					0.506		
N	30	62.9%		–		16	43.2%		–	
Y	11	49.9%		–	–	6	44.4%		–	–
**Postoperative Treatment**			0.036*		0.254			0.020*		0.751
N	25	71.9%		1		16	53.2%		1	
Y	16	36.1%		2.70 (0.49–14.9)		6	16.7%		1.27 (0.30–5.41)	

CI, confidence interval; HR, hazard ratio; p, pathological; ALI, angiolymphatic invasion; N, no; Y, yes.

*P < 0.05.

^a^pN2 and pStage III–IV status were linearly correlated covariates.

Seventeen patients (41.5%) died during the follow-up period, including 5 patients (29.4%) with non-cancer related diseases. The 3- and 5-year OS rates were 70.4% and 57.0%, respectively. The univariate and multivariate analyses of OS are presented in Table 2 with detailed information provided in S2 Table. Age <65 years, female gender, a small tumor size, and the absence of ALI were associated with a better OS. Disease stage, pT status, and pN status influenced OS, whereas tumor grade did not (P = 0.170). In the multivariate analysis, age, tumor size, pT status, and pN2 status were independent prognostic factors for OS.

**Table 2:** Univariate and multivariate analyses of prognostic factors influencing overall survival (OS) after surgical resection in all patients combined (n = 41) and in elderly patients (n = 22).

Factor	All Patients Combined					Elderly Patients (>65 y)				
	Univariate			Multivariate		Univariate			Multivariate	
	n	5-y OS	P-value	HR (95% CI)	P-value	n	5-y OS	P-value	HR (95% CI)	P-value
**Age (y)**			0.007*	1.17 (1.06–1.30)	0.003*					
≤65	19	73.8%				–	–	–	–	–
>65	22	41.5%				–	–		–	
**Sex**			0.032*		0.303			0.337		
M	30	49.2%		1		21	38.0%		–	
F	11	77.8%		0.34 (0.04–2.67)		1	100.0%		–	–
**Tumor Size (cm)**			0.016*		0.008*			0.184		
≤3	25	72.7%		1		14	51.9%		–	
>3	16	29.3%		7.27 (1.68–31.4)		8	19.0%		–	–
**pT Status**			0.020*					0.004*		
T1	13	90.9%		1		6	83.3%		1	
T2	24	44.0%		7.35 (1.29–41.7)	0.024*	15	21.9%		6.26 (1.31–30.0)	0.022*
T3–4	4	25.0%		21.0 (1.16–381.0)	0.040*	1	0.0%		42.40 (2.56–703.4)	0.009*
**pN Status**			<0.001*					0.011*		
N0	26	72.8%		1		16	54.8%		1	
N1	6	80.0%		3.87 (0.27–55.7)	0.320	1	100.0%		–	
N2^a^	9	0.0%		21.1 (3.79–117.6)	<0.001*	5	0.0%		2.55 (0.65–9.98)	0.178
**pStage**			<0.001*					0.012*		
I	21	76.0%		1		13	60.6%		1	
II	11	70.7%		1.17 (0.18–7.68)	0.872	4	37.5%		1.19 (0.36–10.2)	0.448
III–IV^a^	9	0.0%		21.1^a^ (3.79–117.6)	<0.001*	5	0.0%		2.55^a^ (0.65–9.98)	0.178^a^
**Tumor Grade**			0.170					0.154		
Low	10	66.7%		–	–	1	0.0%		–	–
High	31	53.2%		–		21	43.7%		–	
**ALI**			0.002*		0.154			0.012*		0.576
N	30	69.5%		1		16	56.6%		1	
Y	11	15.9%		2.50 (0.71–8.83)		6	0.0%		1.55 (0.33–7.27)	

CI, confidence interval; HR, hazard ratio; M, male; F, female; p, pathological; ALI, angiolymphatic invasion; N, no; Y, yes.

*P < 0.05.

^a^pN2 and pStage III–IV status were linearly correlated covariates.

**Fig 2:**
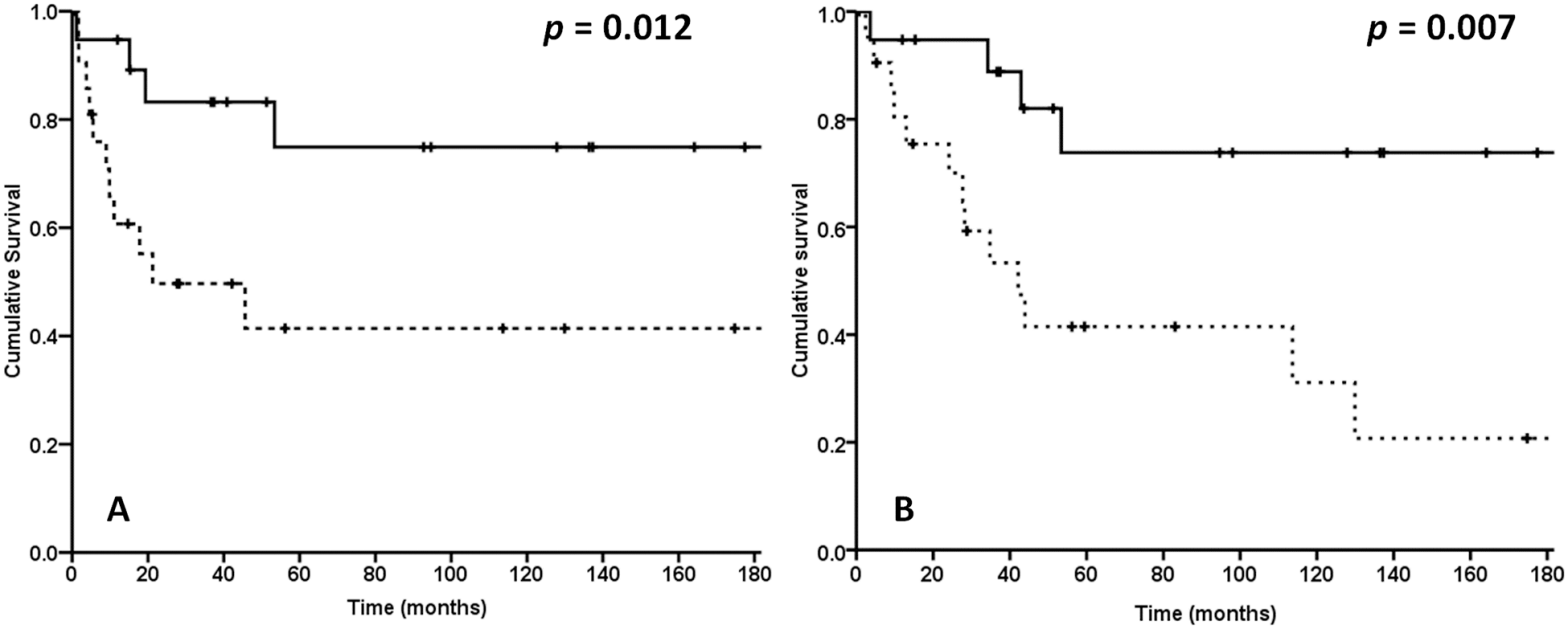
Kaplan-Meier plots. (Left) Disease-free survival and (Right) overall survival in pulmonary mucoepidermoid carcinoma patients (n = 41) stratified by age. Patients in the ≤65 years age group (n = 19) are represented by the solid line and patients in the >65 years (“elderly”) age group (n = 22) are represented by the dashed line. A P < 0.05 was considered statistically significant.

Twenty-two patients (53.7%) were >65 years of age. These patients were considered elderly. The remaining 19 patients (46.3%) were ≤65 years of age. Both DFS and OS rates differed between the older and younger patients as shown in Fig 2A and 2B.

The mean age of the elderly patients was 75.9 years. Twenty-one patients (95.5%) were male and one patient (4.5%) was female. Sixteen patients (72.7%) had a history of smoking. Cough remained the most common presenting symptom (n = 11 patients; 50.0%). Six patients (27.3%) exhibited abnormal bronchoscopic findings. Seventeen patients (77.2%) had a preoperative diagnosis. However, only 4 patients had a precise diagnosis of PMEC. Fifteen patients (68.2%) underwent open thoracotomy. Lobectomy was performed in 19 patients (86.4%) and 3 patients (13.6%) underwent wedge resection. The median tumor size was 3.0 (range, 1.8–9.0) cm. Six patients (27.3%) had pT1, 15 patients (68.2%) had pT2, and one patient (4.5%) had pT3 lesions. Sixteen patients (72.8%) had pN0, one patient (4.5%) had pN1, and 5 patients (22.7%) had pN2 disease. Thirteen patients (59.1%) had pStage I, 4 patients (18.2%) had pStage II, 4 patients (18.2%) had pStage III, and one patient (4.5%) had pStage IV disease. Only one patient (4.5%) had a low-grade tumor; the remaining 21 patients (95.5%) had high-grade tumors. ALI was present in 6 patients (27.3%) and pleural invasion was observed in 12 patients (54.5%). Six patients (27.3%) received postoperative adjuvant treatment.

Among the 22 elderly patients, 12 patients (54.5%) experienced tumor relapse. The 3- and 5-year DFS rates were 49.7% and 41.4%, respectively. The univariate and multivariate analyses of DFS are presented in Table 1 with detailed information provided in S1 Table. Postoperative treatment and pT status influenced DFS, but only pT status was an independent prognostic factor for DFS. Thirteen patients (59.1%) died during the study period, including 4 patients (30.8%) with non-cancer related diseases. The 3- and 5-year OS rates were 53.3% and 41.5%, respectively. The univariate and multivariate analyses of OS are presented in Table 2 with detailed information provided in S2 Table. Disease stage, pT status, pN status, and ALI influenced OS, whereas tumor grade and tumor size did not. In the multivariate analysis, pT status was the only independent prognostic factor for OS in elderly patients.

## Discussion

PMEC was first reported by Smetana et al. [20] in 1952. PMEC accounts for approximately 0.1–0.2% of all lung malignancies [2, 7, 8] and its incidence is increasing according to recent reports [16, 17, 21, 22]. In our series, the incidence of PMEC was similar to that reported by Lee et al., but was higher than that of another report [16]. A possible explanation may relate to the different characteristics of the patient population in our hospital.

Most researchers assume that PMEC originates from the submucosal glands of the trachea and bronchus, which are anatomically centrally located [2, 3]. This is reasonable given that cough is a common presenting symptom and tumors are typically centrally located on imaging studies. In our study, cough was the most frequent presenting symptom followed by hemoptysis. These findings are comparable to those of several other studies [3, 11–13, 15, 22–24], but are different from the results of Yousem et al. [9]. Our study also demonstrated that approximately 40% of patients had abnormal bronchoscopy findings that suggested that the tumor was centrally located, as in several previously published studies [12, 13, 15]. However, only 4 patients had an accurate preoperative diagnosis of PMEC from bronchoscopic biopsy, which represented a lower proportion than that of previous reports [11, 16, 17, 23, 24].

The morphology of PMEC may mimic other types of non-small cell carcinoma, such as adenocarcinoma and adenosquamous carcinoma. Preoperative diagnosis therefore can be challenging in small size of biopsy specimens. Immunohistochemical stains may be helpful in the differential diagnosis. For example, lung adenocarcinomas are frequently positive for TTF-1 and napsin, but PMEC are negative [14]. The distinction between adenosquamous carcinoma and PMEC is more problematic, especially for high grade tumors. It was recently reported that adenosquamous carcinoma can harbor mucoepidermoid carcinoma-like component, which further increased the complexity of differential diagnosis [25]. In these difficult cases, molecular analysis for gene rearrangement of mammalian mastermind-like 2 (MAML2) could be helpful. This genetic change is found in 77% to 100% of PMEC, but not in adenosquamous carcinoma [26, 27].

PMEC is usually regarded as a low-grade malignancy and is associated with significantly better long-term outcomes than non-small cell lung cancer [3]. However, Turnbull et al. [2] reported that the maximum survival time after treatment in PMEC patients was only 18 months, because all 12 patients had high-grade tumors. With the exception of tumor grade, the factors influencing survival were largely undetermined, because most series only included a relatively small number of patients. Recently, several studies [3, 11, 15, 16, 21, 28] have reported DFS and OS rates of >70%. The survival rates in our series were lower than those reported in previous studies [3, 11, 15, 16, 21, 28]. A possible explanation for this discrepancy may relate to differences in the patient characteristics and treatment methods in the study population.

Tumor grade has been considered an important prognostic factor for patients with PMEC. Since Yousem and Hochholzer [9] classified PMEC as low-grade or high-grade based on histological pattern, tumor grade has always been considered important in studies of PMEC. In the majority of series, the authors concluded that patients with low-grade tumors have a better survival rate than those with high-grade tumors despite the fact that no statistical comparisons have been performed. Furthermore, Zhu et al. [15] demonstrated that tumor grade was an independent prognostic factor in multivariate analysis. In our study, tumor grade did not influence DFS or OS. The primary reason for the differing results is that patients with low-grade tumors are associated with poorer survival rates in our study (DFS: 66.7%, OS: 66.7%) compared to previous studies [8–13, 15–18, 21, 23–24, 28] with survival rates of ≥80%, or even 100%. However, the survival rates of patients with high-grade tumors in our study were comparable to those reported in other studies [10, 11, 15, 23].

Spencer [29] reported that the majority of mucoepidermoid bronchial tumors occur in younger patients, while malignant mucoepidermoid tumors only occur in elderly patients, suggesting that the malignant potential of the tumor might be related to age. PMECs, on the other hand, occur over a wide age range and age is frequently considered a factor that can influence survival. The majority of studies [10–12, 28] have shown that younger patients are associated with better DFS and OS rates. Our study also found that a younger age was a better independent prognostic factor for both DFS and OS.

Several studies [11, 13, 24] have suggested that younger patients are more likely to have low-grade PMECs and our study demonstrated similar results. Furthermore, although the numbers of patients were limited in previous studies [8–12, 21, 22, 30], it is easy to observe the trend that the average age of the patients was related to the tumor grade. In our study, we focused on elderly patients of >65 years of age and analyzed the prognostic factors influencing DFS and OS. In contrast to the analysis of all patients combined, pT status was the only independent prognostic factor for both DFS and OS in elderly patients.

Tumor size and ALI have been studied in various types of cancer [31–33], but have seldom been discussed in relation to PMEC. This may be due to the low incidence of PMEC. Our study demonstrated that a tumor size of <3 cm was associated with a better DFS and OS, and tumor size was an independent prognostic factor in the multivariate analysis. Huo et al. [12] reported similar findings. However, tumor size did not influence DFS or OS in elderly patients. ALI was a prognostic factor for OS, but not DFS, in our series, including elderly patients. Our findings are similar to those of previous studies [32, 33] that focused on patients with Stage I non-small cell lung cancer.

Although Huo et al. [12] concluded that pN status did not influence survival, pN status and disease stage have been reported to be prognostic factors of PMEC in many other studies [9–11, 17, 21, 22, 30]. In our series, we observed similar findings in our analysis of all patients combined, but not in our analysis of elderly patients. Other factors (e.g., pT status) were also found to be independent prognostic factors for DFS and OS in our study. Moreover, in elderly patients, pT status was the only independent prognostic factor that influenced both DFS and OS.

Our study had several limitations including its retrospective nature and limited sample size. The management of some N2 patients may not follow the current treatment guidelines. Future prospective studies involving larger patient cohorts are, therefore, needed to confirm our findings.

## Conclusions

The prognostic factors for PMEC identified in this study differ from those in other studies. Principally, tumor grade did not influence either DFS or OS. Age, tumor size, and pathological factors were independent prognostic factors for DFS and OS. Prognostic factors for elderly PMEC patients differed from those for all PMEC patients combined. In elderly patients, pT status was the only prognostic factor for both DFS and OS.

## Supporting information

S1 Table Detailed univariate and multivariate analyses of prognostic factors influencing disease-free survival (DFS) after surgical resection in all patients combined (n = 41) and in elderly patients (n = 22). (DOCX)

S2 Table Detailed univariate and multivariate analyses of prognostic factors influencing overall survival (OS) after surgical resection in all patients combined (n = 41) and in elderly patients (n = 22). (DOCX)
