# Supplementary material for: Surgical outcomes of pulmonary mucoepidermoid carcinoma: A review of 41 cases
Source: PLoS One. 2017 May 2;12(5):e0176918. doi: 10.1371/journal.pone.0176918 (PMC5413008; doi:10.1371/journal.pone.0176918)
Supplement: S1 Table — (DOCX) [file pone.0176918.s001.docx]

**S1 Table. Detailed Univariate and Multivariate Analyses of Prognostic Factors Influencing Disease-Free Survival (DFS) After Surgical Resection in All Patients Combined (n = 41) and in Elderly Patients (n = 22).**

| **Factors** | | **All Patients Combined** | | | | | **Elderly Patients (>65 y)** | | | | |
| --- | --- | --- | --- | --- | --- | --- | --- | --- | --- | --- | --- |
|  |  | **Univariate** | | | **Multivariate** | | **Univariate** | | | **Multivariate** | |
|  |  | **n** | **5-y DFS** | **P-value** | **HR (95% CI)** | **P-value** | **n** | **5-y DFS** | **P-value** | **HR (95% CI)** | **P-value** |
| **Age (y)** | | |  | 0.012^*^ | 1.10 (1.03–1.18) | 0.003^*^ |  |  |  |  |  |
|  | ≤65 | 19 | 74.9% |  | – |  | – | – | – | – | – |
|  | >65 | 22 | 41.4% |  | – |  | – | – |  | – |  |
| **Sex** | | |  | 0.140 |  |  |  |  | 0.561 |  |  |
|  | M | 30 | 54.5% |  | – | – | 21 | 44.0% |  | – | – |
|  | F | 11 | 68.6% |  | – |  | 1 | 0.0% |  | – |  |
| **Smoking Status** | | |  | 0.205 | – |  |  |  | 0.684 |  |  |
|  | N | 20 | 64.3% |  | – |  | 6 | 44.4% |  | – |  |
|  | Y | 21 | 52.1% |  | – | – | 16 | 42.0% |  | – | – |
| **Tumor Location** | | |  | 0.220 |  |  |  |  | 0.154 |  |  |
|  | R | 22 | 75.9% |  | – | – | 13 | 65.6% |  | – | – |
|  | L | 19 | 40.1% |  | – |  | 9 | 13.9% |  | – |  |
| **Surgical Method** | | |  | 0.934 |  |  |  |  | 0.778 |  |  |
|  | OT | 30 | 58.6% |  | – | – | 15 | 44.4% |  | – | – |
|  | VATS | 11 | 58.3% |  | – |  | 7 | 30.0% |  | – |  |
| **Surgical Resection** | | |  | 0.593 |  |  |  |  | 0.395 |  |  |
|  | PNT+B | 5 | 60.0% |  | – | – | 0 | – |  | – | – |
|  | LB | 31 | 63.7% |  | – |  | 19 | 45.8% |  | – |  |
|  | WR | 5 | 25.0% |  | – |  | 3 | 0.0% |  | – |  |
| **Tumor Size (cm)** | | |  | 0.005^*^ |  | 0.005^*^ |  |  | 0.082 |  | 0.076 |
|  | ≤3 | 25 | 72.8% |  | 1 |  | 14 | 49.5% |  | 1 |  |
|  | >3 | 16 | 32.8% |  | 6.53 (1.78–24.0) |  | 8 | 28.6% |  | 3.17 (0.89–11.4) |  |
| **pT Status** | | |  | 0.054 |  |  |  |  | <0.001^*^ |  |  |
|  | T1 | 13 | 82.5% |  | 1 |  | 6 | 66.7% |  | 1 |  |
|  | T2 | 24 | 53.5% |  | 3.22 (0.49–21.0) | 0.221 | 15 | 37.7% |  | 7.72 (1.47–40.5) | 0.016^*^ |
|  | T3–4 | 4 | 25.0% |  | 22.5 (1.10–461.9) | 0.043^*^ | 1 | 0.0% |  | 93.9 (4.25–2073.6) | 0.004^*^ |
| **pN Status** | | |  | 0.021^*^ |  |  |  |  | 0.331 |  |  |
|  | N0 | 26 | 68.5% |  | 1 |  | 16 | 46.5% |  | – |  |
|  | N1 | 6 | 66.7% |  | 5.81 (0.77–44.1) | 0.089 | 1 | 0.0% |  | – |  |
|  | N2^a^ | 9 | 0.0% |  | 12.0 (2.63–54.7) | 0.001^*^ | 5 | 40.0% |  | – | – |
| **pStage** | | |  | 0.015^*^ |  |  |  |  | 0.289 |  |  |
|  | I | 21 | 69.8% |  | 1 |  | 13 | 49.1% |  | – |  |
|  | II | 11 | 63.6% |  | 2.12 (0.33–13.8) | 0.432 | 4 | 25.0% |  | – |  |
|  | III–IV^a^ | 9 | 0.0% |  | 12.0 (2.63–54.7) | 0.001^*^ | 5 | 40.0% |  | – | – |
| **Tumor Grade** | | |  | 0.286 |  |  |  |  | 0.146 |  |  |
|  | Low | 10 | 66.7% |  | – | – | 1 | 0.0% |  | – | – |
|  | High | 31 | 54.9% |  | – |  | 21 | 43.6% |  | – |  |
| **ALI** | | |  | 0.145 |  |  |  |  | 0.506 |  |  |
|  | N | 30 | 62.8% |  | – |  | 16 | 43.2% |  | – |  |
|  | Y | 11 | 49.9% |  | – | – | 6 | 44.4% |  | – | – |
| **Pleural Invasion** | | |  | 0.335 |  |  |  |  | 0.704 |  |  |
|  | N | 24 | 63.0% |  | – |  | 10 | 32.0% |  |  |  |
|  | Y | 17 | 50.5% |  | – | – | 12 | 51.1% |  | – | – |
| **Postoperative Treatment** | | |  | 0.036^*^ |  | 0.254 |  |  | 0.020^*^ |  | 0.751 |
|  | N | 25 | 71.9% |  | 1 |  | 16 | 53.2% |  | 1 |  |
|  | Y | 16 | 36.1% |  | 2.70 (0.49–14.9) |  | 6 | 16.7% |  | 1.27 (0.30–5.41) |  |

ALI, angiolymphatic invasion; B, bilobectomy; CI, confidence interval; F, female; HR, hazard ratio; L, left; LB, lobectomy; M, male; N, no; OT, open thoracotomy; p, pathological; PNT, pneumonectomy; R, right; VATS, video-assisted thoracoscopic surgery; WR, wedge resection; Y, yes.

^*^P < 0.05.

^a^pN2 and pStage III–IV status were linearly correlated covariates.
