# Supplementary material for: Surgical outcomes of pulmonary mucoepidermoid carcinoma: A review of 41 cases
Source: PLoS One. 2017 May 2;12(5):e0176918. doi: 10.1371/journal.pone.0176918 (PMC5413008; doi:10.1371/journal.pone.0176918)
Supplement: S2 Table — (DOCX) [file pone.0176918.s002.docx]

**S2 Table. Detailed Univariate and Multivariate Analyses of Prognostic Factors Influencing Overall Survival (OS) After Surgical Resection in All Patients Combined (n = 41) and in Elderly patients (n = 22).**

| **Factors** | | **All Patients Combined** | | | | | **Elderly Patients (>65 y)** | | | | |
| --- | --- | --- | --- | --- | --- | --- | --- | --- | --- | --- | --- |
|  |  | **Univariate** | | | **Multivariate** | | **Univariate** | | | **Multivariate** | |
|  |  | **n** | **5-y OS** | **P-value** | **HR (95% CI)** | **P-value** | **n** | **5-y OS** | **P-value** | **HR (95% CI)** | **P-value** |
| **Age (y)** | | |  | 0.007^*^ | 1.17 (1.06–1.30) | 0.003^*^ |  |  |  |  |  |
| **Age (y)** | ≤65 | 19 | 73.8% |  |  |  | – | – | – | – | – |
|  | >65 | 22 | 41.5% |  |  |  | – | – |  | – |  |
| **Sex** | | |  | 0.032^*^ |  | 0.303 |  |  | 0.337 |  |  |
|  | M | 30 | 49.2% |  | 1 |  | 21 | 38.0% |  | – |  |
|  | F | 11 | 77.8% |  | 0.34 (0.04–2.67) |  | 1 | 100.0% |  | – | – |
| **Smoking Status** | | |  | 0.114 |  |  |  |  | 0.855 |  |  |
|  | N | 20 | 63.8% |  | – |  | 6 | 41.7% |  | – |  |
|  | Y | 21 | 51.7% |  | – | – | 16 | 41.6% |  | – | – |
| **Tumor Location** | | |  | 0.840 |  |  |  |  | 0.768 |  |  |
|  | R | 22 | 58.7% |  | – | – | 13 | 37.0% |  | – | – |
|  | L | 19 | 54.5% |  | – |  | 9 | 45.7% |  | – |  |
| **Surgical Method** | | |  | 0.734 |  |  |  |  | 0.714 |  |  |
|  | OT | 30 | 56.1% |  | – | – | 15 | 40.0% |  | – | – |
|  | VATS | 11 | 70.0% |  | – |  | 7 | 53.3% |  | – |  |
| **Surgical Resection** | | |  | 0.766 |  |  |  |  | 0.448 |  |  |
|  | PNT+B | 5 | 60.0% |  | – | – | 0 | – |  | – | – |
|  | LB | 31 | 62.5% |  | – |  | 19 | 46.5% |  | – |  |
|  | WR | 5 | 25.0% |  | – |  | 3 | 0.0% |  | – |  |
| **Tumor Size (cm)** | | |  | 0.016^*^ |  | 0.008^*^ |  |  | 0.184 |  |  |
|  | ≤3 | 25 | 72.7% |  | 1 |  | 14 | 51.9% |  | – |  |
|  | >3 | 16 | 29.3% |  | 7.27 (1.68–31.4) |  | 8 | 19.0% |  | – | – |
| **pT Status** | |  |  | 0.020^*^ |  |  |  |  | 0.004^*^ |  |  |
|  | T1 | 13 | 90.9% |  | 1 |  | 6 | 83.3% |  | 1 |  |
|  | T2 | 24 | 44.0% |  | 7.35 (1.29–41.7) | 0.024^*^ | 15 | 21.9% |  | 6.26 (1.31–30.0) | 0.022^*^ |
|  | T3–4 | 4 | 25.0% |  | 21.0 (1.16–381.0) | 0.040^*^ | 1 | 0.0% |  | 42.40 (2.56–703.4) | 0.009^*^ |
| **pN Status** | | |  | <0.001^*^ |  |  |  |  | 0.011^*^ |  |  |
|  | N0 | 26 | 72.8% |  | 1 |  | 16 | 54.8% |  | 1 |  |
|  | N1 | 6 | 80.0% |  | 3.87 (0.27–55.7) | 0.320 | 1 | 100.0% |  | – |  |
|  | N2^a^ | 9 | 0.0% |  | 21.1 (3.79–117.6) | <0.001^*^ | 5 | 0.0% |  | 2.55 (0.65–9.98) | 0.178 |
| **pStage** | | |  | <0.001^*^ |  |  |  |  | 0.012^*^ |  |  |
|  | I | 21 | 76.0% |  | 1 |  | 13 | 60.6% |  | 1 |  |
|  | II | 11 | 70.7% |  | 1.17 (0.18–7.68) | 0.872 | 4 | 37.5% |  | 1.19 (0.36–10.2) | 0.448 |
|  | III–IV^a^ | 9 | 0.0% |  | 21.1 (3.79–117.6) | <0.001^*^ | 5 | 0.0% |  | 2.55 (0.65–9.98) | 0.178 |
| **Tumor Grade** | | |  | 0.170 |  |  |  |  | 0.154 |  |  |
|  | Low | 10 | 66.7% |  | – | – | 1 | 0.0% |  | – | – |
|  | High | 31 | 53.2% |  | – |  | 21 | 43.7% |  | – |  |
| **ALI** | | |  | 0.002^*^ |  | 0.154 |  |  | 0.012^*^ |  | 0.576 |
|  | N | 30 | 69.5% |  | 1 |  | 16 | 56.6% |  | 1 |  |
|  | Y | 11 | 15.9% |  | 2.50 (0.71–8.83) |  | 6 | 0.0% |  | 1.55 (0.33–7.27) |  |
| **Pleural Invasion** | | |  | 0.320 |  |  |  |  | 0.802 |  |  |
|  | N | 24 | 62.1% |  | – |  | 10 | 34.3% |  | – | – |
|  | Y | 17 | 50.0% |  | – | – | 12 | 49.1% |  | – | – |
| **Postoperative Treatment** | | |  | 0.183 |  |  |  |  | 0.179 |  |  |
|  | N | 25 | 66.8% |  | – |  | 16 | 47.0% |  |  |  |
|  | Y | 16 | 41.6% |  | – | – | 6 | 33.3% |  | – |  |

ALI, angiolymphatic invasion; B, bilobectomy; CI, confidence interval; F, female; HR, hazard ratio; L, left; LB, lobectomy; M, male; N, no; OT, open thoracotomy; p, pathological; PNT, pneumonectomy; R, right; VATS, video-assisted thoracoscopic surgery; WR, wedge resection; Y, yes.

^*^P < 0.05.

^a^pN2 and pStage III–IV status were linearly correlated covariates.
